# SURABHI: Self-Training Using Rectified Annotations-Based Hard Instances for Eidetic Cattle Recognition

**DOI:** 10.3390/s24237680

**Published:** 2024-11-30

**Authors:** Manu Ramesh, Amy R. Reibman

**Affiliations:** School of Electrical and Computer Engineering, Purdue University, West Lafayette, IN 47907, USA

**Keywords:** self-training, hard instances, keypoint detection, cattle recognition

## Abstract

We propose a self-training scheme, SURABHI, that trains deep-learning keypoint detection models on machine-annotated instances, together with the methodology to generate those instances. SURABHI aims to improve the keypoint detection accuracy not by altering the structure of a deep-learning-based keypoint detector model but by generating highly effective training instances. The machine-annotated instances used in SURABHI are hard instances—instances that require a rectifier to correct the keypoints misplaced by the keypoint detection model. We engineer this scheme for the task of predicting keypoints of cattle from the top, in conjunction with our Eidetic Cattle Recognition System, which is dependent on accurate prediction of keypoints for predicting the correct cow ID. We show that the final cow ID prediction accuracy on previously unseen cows also improves significantly after applying SURABHI to a deep-learning detection model with high capacity, especially when available training data are minimal. SURABHI helps us achieve a top-6 cow recognition accuracy of 91.89% on a dataset of cow videos. Using SURABHI on this dataset also improves the number of cow instances with correct identification by 22% over the baseline result from fully supervised training.

## 1. Introduction

Keypoints or landmark points detected on objects serve as the foundation for many downstream tasks, including cattle recognition [1], cattle weight estimation [2], activity recognition and tracking [3], sign language translation [4], and object alignment [5]. However, if this foundation is fragile, any downstream task becomes vulnerable to inaccuracies. For instance, for a cattle identification system such as the one in [1] which is contingent on correct detection of keypoints, in the absence of the built-in guardrails, keypoint misplacements would generate the wrong feature vector for the queried individual, resulting in misidentification. Further, misdetected keypoints in a sign language interpretation system such as the one in [4] will result in wrong translation.

In this paper, we propose SURABHI, a method that uses the outputs of subsequent downstream vision tasks and a given keypoint rectifier to strengthen keypoint detection models precisely in their weak points. The goal of SURABHI lies not in optimizing the architecture of keypoint detection models but in finding the best instances to augment the training dataset so that the keypoint detection performance improves upon retraining. These instances obtained by SURABHI are the ones that are ‘hard’ for the model, i.e., the detection model cannot correctly predict keypoint locations for these instances. Further, SURABHI applies a keypoint rectifier to correct these misplaced keypoints to automatically generate correctly annotated self-training instances.

SURABHI differs from traditional pseudo-labeling, which uses the initially trained model to infer on unlabeled data and then uses the inferred labels directly as ground-truth annotations for the unlabeled data. The traditional approach adds such pseudo-labeled data-annotation pairs to the subsequent versions of the training datasets. These pseudo-labeled samples are those that the detection model predicts with high confidence. However, there is no built-in way to determine automatically if these pseudo-labeled examples are hard or easy. Specifically, we consider easy instances to be those that the model can get right already, namely, all annotated instances that are directly generated by the keypoint detection model and are above a certain confidence threshold determined by a set of rules.

SURABHI is designed to use hard instances for retraining and ignore easy instances. This has two evident advantages:

1. The annotations do not require human effort, as they are performed by the machine.
2. These hard instances are the exact weak points of the keypoint detection model, so these automatically generated additional samples fill the gaps in the original training dataset.

Our intuition is that training the keypoint detection models on these easy instances will not be of much help. This is like students trying to ace a math final exam, not being able to improve their chances on the exam by practicing extensively on the kind of questions they did well on in the practice tests. Putting in many hours solving the kind of problems they had no difficulty with on the practice tests (easy instances) will only make them complacent and perhaps fail the finals. However, should they practice more on the kind of questions they did not do well on (hard instances), they will definitely improve their odds on the final exam. In the same sense, the keypoint detection model is strengthened by learning from those annotations it missed that could be corrected by a keypoint rectifier, thereby learning from instances on which it initially performed poorly. Hard instances were also prioritized in successful techniques such as the focal loss [6] and HEXA [7].

By improving keypoint detection accuracy, SURABHI has the potential to improve the performance of downstream tasks such as cattle recognition [1]. Since cattle recognition serves as a foundation for tracking cattle health, behavior patterns, milk production, etc., improvements in keypoint detection accuracy are likely to also improve the performance of these downstream tasks.

The development of the SURABHI methodology was motivated by our need to improve the performance of our eidetic cattle recognition system in [1]. This cattle recognition system aligns the top view of a cow into a canonical pose using keypoints detected on their backs to assist identification. (More details are in Section 3).

In the cattle recognition system in [1], we noted that the keypoint detector, keypoint-RCNN [8], was a weak link as it often misplaced keypoints. In that work, a component that checks for keypoint correctness was designed to prevent the system from using misplaced keypoints for identification. This reduced misidentification of the cow instances. However, recovering such hard instances by rectifying the misplaced keypoints and learning from these mistakes must further improve the identification performance. For this, we employ SURABHI in conjunction with a keypoint rectifier.

Note that SURABHI is agnostic to the underlying keypoint detector, and with a given keypoint rectifier, it can be applied to any keypoint detector. In this paper, we use the terms ‘rectification’ and ‘correction’ interchangeably, and they are both used in the sense of eliminating errors in keypoint detection. Additionally, for brevity, we shall refer to the keypoint detector as ‘model’ and the end-to-end Eidetic Cattle Recognition system of which this ‘model’ is a part as ‘cow recognition system’ or sometimes just ‘system’.

The key contributions we make in this paper are as follows:

1. We show that keypoint detection models will become more accurate when trained on additional instances that are hard for them, rather than when trained on additional easy instances.
2. We show that improving keypoint detection performance using hard instances potentially improves the performance of the subsequent downstream tasks. For this, we demonstrate a more accurate eidetic cattle recognition system that incorporates a keypoint rectifier and the SURABHI training methodology.

We first review related works in Section 2. In Section 3, we explain the updated cow recognition system, together with the strategy for estimating and correcting misplaced keypoints. A more in-depth explanation of hard instances, their need, and how they are used to improve keypoint detection is also in the same section. Then in Section 4, we elaborate on all our datasets, and in Section 5, the evaluation criteria, strategy, and metrics used in our experiments. Section 6 has the details of all the experiments we conducted, in which we consider both half-day and whole-day experiments. The former allows exploration of SURABHI and its role in improving cow recognition. The latter enables a direct performance comparison of the cow recognition system with SURABHI presented in this paper to that of [1]. The results of these experiments are also discussed in the same section.

## 2. Related Works

In this section, we explore the techniques available for semi-supervision or self-training and self-supervision for keypoint detection and explain how our method differs. We then present a short literature survey on methods that detect keypoints on cows. After that, we present related works in the domain of cattle recognition with computer vision and justify why our technique [1] that uses keypoints detected on top of cows is more apt for the task.

### 2.1. Semi- and Self-Supervision for Keypoint Detection

Many methods [9,10,11,12,13,14] propose online self-training, which uses a mix of images with and without keypoint annotations in each training batch. Among these, [9] optimizes unsupervised losses such as those that measure deviation in transformation equivariance of keypoint locations, transformation invariance of keypoint feature vectors, and semantic consistency of the keypoint region, along with supervised landmark loss in each batch. Some methods learn to detect keypoints better by utilizing weaker annotations such as image classification labels [10], edge linkages [11], and object bounding boxes [12]. Other methods enforce keypoint consistency using constraints such as epipolar divergence in multiple views [13] and affinity between keypoint regions in transformed image pairs to simulate nearby frames of a video [14]. The approach in [14] uses the self-annotated training instances only to train the keypoint correspondence network; they are not used to improve the performance of the keypoint detector.

Unlike the above methods, our method does not self-train the models ‘online’, i.e., it does not combine images with and without annotations in a training batch. In that sense, the self-training in our method is conducted ‘offline’, because we generate annotated hard instances first and then retrain the model on a dataset that includes those hard instances to improve the keypoint detection accuracy. Every hard instance in our training batch has keypoint annotations that come from a stand-alone keypoint rectifier. Also, our approach does not alter the keypoint detector model architecture in any way. Instead, it finds instances that are known weak points of the initial model and uses these to improve the performance of the same keypoint detection model architecture.

A keypoint detector agnostic plug-and-play technique for improving keypoint detection accuracy by using Procrustes analysis [15] to regularize pseudo-labels to fit a shape model is described in [16]. This method seems very apt to source hard instances for SURABHI. However, this Procrustes technique is not suitable for our example case of correcting top view cow keypoints. The Procrustes technique finds the best linear transformation that needs to be applied to one set of keypoints so that they closely match a template set. Any misplaced keypoint could be identified by finding the keypoint that deviates the most from the template, but the problem here arises from the fact that all operations involved in the Procrustes analysis are *linear*. This is not suitable for bodies of cows in the top view because they are non-linearly deformable. The cow, if bent along the spine, would lead to miscorrection of the spine keypoints if a linear interpolation method is used. So, in the keypoint rectification process, the system fits a second-order polynomial curve along the correct spine points and places the misplaced one at a point on this curve. The Procrustes technique cannot achieve this.

### 2.2. Cattle Keypoint Detection

We now present a brief survey of methods in literature that detect keypoints on cows.

Keypoints are detected on cattle mostly in the side views for estimating their pose [17,18,19], body sizes [20], and motion behaviors [21]. Methods to detect keypoints on cows in the side view are discussed in [22] with the aim of obtaining gait features. A method to obtain refined keypoints on the side view of cows has been discussed in [23], and these keypoints were used for visual cow weight estimation in [2].

While the above methods deal with keypoint detection in the side view of cows, [24] describes a method that detects seven keypoints on cows in their top view and uses distances between keypoints as features for identifying cows with no visible coat patterns, demonstrating performance on a set of 25 cows. Our approach [1], designed for cows with visible coat patterns, also detects cow keypoints in the top-view scenes and uses these keypoints to align cows in different poses into a canonical pose to aid their individual identification. Further, we verify its scalability by evaluating it on a set of nearly 150 cows.

### 2.3. Cattle Recognition Using Computer Vision

Computer vision methods for cattle recognition outperform other methods such as ear-tagging, ear-notching, branding, and RFID tags in terms of speed, scalability, and maintainability. So, here we discuss computer vision-based methods available for cattle recognition. In the process, we also explain the advantages of our method [1] over the other listed methods.

Different methods have been proposed to identify cows using computer vision. Some use computer vision techniques to differentiate between muzzle prints of individual cows [25,26,27]. Such techniques neither provide instantaneous identification of cows nor can they be automated. Other methods detect cow faces and use them to identify cows [28,29,30]. These methods inherently learn to recognize the black and white pattern on the faces of Holstein cows. We prefer to identify Holstein cows from their back coat patterns; the back has a larger area than the face, so it can exhibit a larger variation in appearance. Some recent methods [31] perform second-order identification; that is, they depend on primary modes of identification such as ear tags. The method in [31] works by first detecting ear tags in images of cows and then identifying these cows using Optical Character Recognition (OCR) to read the cow IDs on these tags.

A common hurdle for identifying cows with faces or ear tags is that they are not all oriented in the same way in a general setting. This renders such methods useless when the cows do not face the cameras. To tackle such issues, methods such as [31] incorporate computational overheads in the form of a ‘When-To-Read’ module to find the correct frame for identifying a cow. Identifying cows from their backs in the top view has a built-in advantage that cows are always aligned towards the camera when standing beneath it. So, any frame can be used to identify the cows. Also, a camera placed high above can be used to identify multiple cows in the top view simultaneously since all their backs will be visible and aligned upwards. Similarly, our prior work [1] is designed to use coat patterns in top view to identify individual cows, which also enables it to detect and identify more than one cow in a scene.

The methods in [32,33,34,35,36,37,38] use coat patterns on the cows to identify individual cows. Of these, ref. [36] uses a side view, and the rest use the top view of cows for identification. As with using faces or ear-tag OCR for identification, using the side view for identification poses a similar problem that the cows are not always oriented the same way towards the camera. For an identification algorithm to function effectively on a large set of cow identities, the identification model must be trained on many sample images of cows in different possible orientations. Using the top view constrains the orientation of the cows and limits the amount of required image samples.

The methods in [32,35] use deep neural network classifiers for identification. So, as the number of cows in the herd increases or decreases, the output layer of the network needs to be modified to accommodate the exact number of cow identity classes. The network should also be retrained when new cows are added to the barn or when older ones are removed. The SVM and QDA classifiers used in [33] need to be retrained on every change to the set of individual cows in the herd. The method in [34] uses deep metric learning for cow identification. Although it can identify if a cow is from outside the set of individuals used for training, it cannot learn to identify new cows without undergoing retraining. The method in [38] also uses metric learning, along with cluster analysis and self-supervision for identification. The paper [38] demonstrates that its method generalizes to a smaller set of unseen cows without retraining. However, it does not demonstrate how the method would perform when the set of unseen individuals is united with the set of training individuals. In other words, there is no proof that the model would perform well without retraining when new identities need to be learnt. Note that retraining the models requires time, energy, and many training samples.

Unlike the above methods, our method [1] does not solve individual identification as a multi-class classification problem with a predetermined number of classes. It instead converts instances of cows into vectors in a predetermined binary feature space. Even when individuals are added or removed from the herd, the point associated with each individual cow in this feature space is invariant. Therefore, updating the recognition system when the composition of the herd changes is as simple as adding or deleting points directly in this feature space. Hence, unlike the other methods, changes to the cow database can be registered almost instantaneously, without needing to retrain the cow recognition model.

One interesting approach uses temporal features obtained from the motion of cows, along with spatial appearance features, to identify them [39]. This method applies CNN and BiLSTM to solve cattle identification as a multi-class classification problem, implying again that the model must be retrained every time the composition of the herd changes.

The method in [40] aims to circumvent the need for many labeled training images for their CNN-based cow identification model. Although they need fewer training instances for learning cow identities, they cannot avoid retraining. Additionally, this method too solves cattle identification as a multi-class classification problem. So, even if a single cow is added or deleted from the herd, their classification head needs to change, and the model needs to be retrained. As opposed to this method, since ours [1] uses keypoint alignment to bring cows in any orientation, location, or size to a canonical pose before identification, only one training sample is sufficient to learn the identity of a cow.

## 3. Materials and Methods

In this section, we describe the basic concept of SURABHI and then explain its application to the context of improving cattle recognition accuracy.

**Figure 1:**
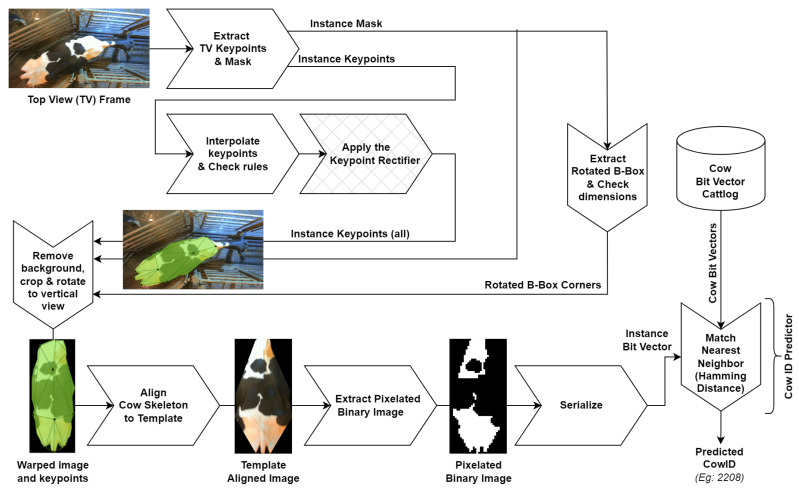
Updated cow recognition system block diagram with keypoint rectifier block (highlighted with cross-hatching).

We first explain the intuition behind choosing hard instances over traditional pseudo-labeled instances for self-training the keypoint detector in Section 3.1. Next, in Section 3.2 we explain what we mean by ‘hard instances’ among the different types of instances available for this self-training. We then move to the domain of SURABHI for eidetic cattle recognition. In this context, we first review the operation of the cattle recognition system in [1] in Section 3.3. The cattle recognition system from [1] is now updated to have a keypoint rectifier block as shown in Figure 1. This keypoint rectifier corrects misplaced keypoints from the keypoint detector, and these instances with rectified keypoints are used as hard instances in SURABHI to loop back and self-train the keypoint detector. In Section 3.4, we discuss what hard instances are in the context of eidetic cattle recognition while also stating and justifying our preference in choosing instances where all keypoints are visible to self-train the cow keypoint detector. Implementation details of the keypoint rectifier used to generate these hard instances are in Section 3.5. This keypoint rectifier also helps cow recognition by correcting misplaced keypoints during inference.

### 3.1. Self-Training Using Hard Instances

In the general setting, we use a small, human-annotated seed dataset to train a default keypoint detection model. We then apply this default model to a large set of unannotated images of objects in the same setting and collect instances with misplaced keypoints. We apply a keypoint rectifier to these instances with misplaced keypoints to create keypoint-rectified instances. These keypoint rectified instances are our ‘hard instances’. We then add the images and annotations of these hard instances to the initial seed dataset to create an expanded dataset. We use this expanded dataset to retrain the keypoint detector model. Since we use annotations produced by the system itself to train the keypoint detector model, this becomes self-training.

In the above process, to judge the correctness of keypoint detection, we do not use the keypoint detection confidence computed by the deep-learning-based keypoint detector. Instead, we consider a confidence metric that is measured by the keypoint rectifier and is correlated to the number of misplaced keypoints. In this manner, SURABHI differs from self-training with traditional pseudo labels.

Self-training the models with traditional pseudo labels would mean accepting as correct whatever the keypoint detector produces with high confidence, even when the detector predicts keypoints in all the wrong locations. Training the keypoint detection model on such instances will scar the model. On the other hand, those pseudo-labeled instances with correctly detected keypoints are still ‘easy instances’ because the detector already knows how to detect them correctly. Intuitively, self-training on such instances should therefore have a low impact.

To judge the correctness of keypoints and further to rectify them, we prefer a rectifier that uses shape priors. We will define these shape priors relative to the positions of keypoints, leveraging object symmetry and object deformability models. SURABHI uses such a rectifier to find instances on which the keypoint detector struggles initially and retrains the detector on them to fortify it exactly in its weak points.

### 3.2. Hard Instances

We define a ‘hard instance’ in general to be an instance in which the keypoints are not in their correct locations in the output of the keypoint detector but are so after undergoing keypoint rectification by a separate keypoint rectifier. Since the keypoint detection model struggles to get the keypoint locations correct on its own, we deem these instances to be ‘hard’ for the detector model.

The procedure of correcting keypoints was developed because the keypoint detector does not always detect them in the right locations. Making the keypoint detector perform better would improve the accuracy of downstream tasks. The plan here is to use these hard instances to retrain the keypoint detector to seek this performance improvement. The intuition behind this is that training on instances that the model is known to fail on should reinforce the model exactly at its weak points. But hard instances are not the only available options for this retraining. All instances on which the model performs poorly could be painstakingly annotated by humans. But, to reduce human effort, we use annotations generated by the machine whenever it can correct the mistakes of the model.

**Figure 2:**
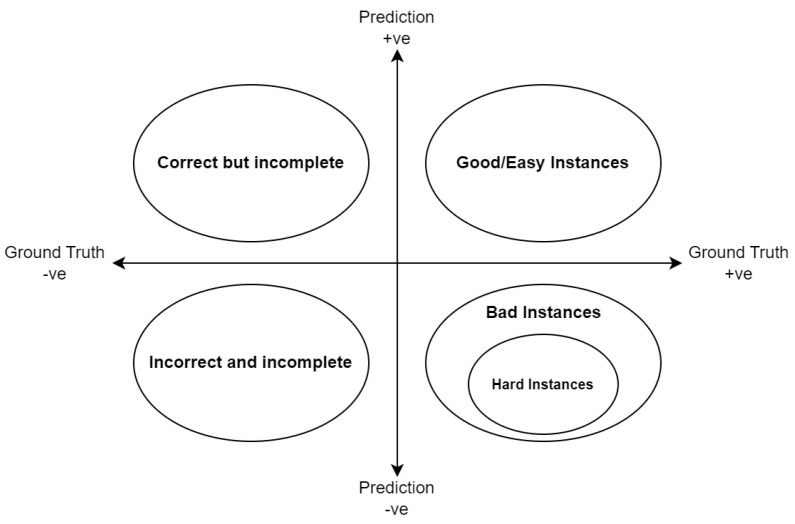
Abstract representation of good/easy, bad, and hard instances using Cartesian quadrants with Venn diagrams inside them.

The different types of machine-annotated instances, including ‘hard instances’, can be described with the help of an abstraction shown in Figure 2. In the figure, ground truth being negative (−ve)We prefer training the keypoint detectors only on images where the objects of interest are completely visible so that the accuracy of the downstream tasks is better. Therefore, we do not use images without a complete set of keypoints visible on the object in the scene for retraining the keypoint detectors. Here, a complete set of keypoints is the set of all detectable keypoints for an instance of that type.

In the same figure, ground truth being positive (+ve)All instances where the keypoint detector predicts the keypoints in the correct places are ‘good instances’, which can be directly used to augment the training dataset. We call them ‘easy instances’ as the detector had no difficulty in getting keypoints right. So,

(1) EasyInstancesSet=GoodInstancesSet

All instances where the keypoint detector, with help as needed from the keypoint interpolator (see Figure 1), detects a complete set of keypoints but fails to detect them in the right places are termed ‘bad instances’. They cannot be directly used to augment the training dataset. However, these are exactly the weak points of the keypoint detector model and need strengthening. Some of these instances with bad keypoint annotations, upon being subjected to our keypoint rectifier, can in fact turn out to be useful if all misplaced keypoints are shifted to their correct locations. These are the ‘hard instances’. These instances, by virtue of being correct, can be used to reinforce the model via retraining. Some other bad instances are just too hard for the keypoint rectifier. They cannot be machine corrected and hence are not used for training. So,

(2) HardInstancesSet⊆BadInstancesSet

In summary, good or easy instances are instances in which the complete set of keypoints from the detector model are in their correct locations. Bad instances are those instances with misplaced keypoints. These bad instances could be used to reinforce the model through retraining given the correct annotations. Hard instances are machine-corrected bad instances.

Next, we move to the domain of applying SURABHI for cow recognition.

### 3.3. Review of Eidetic Cow Recognition System

**Figure 3:**
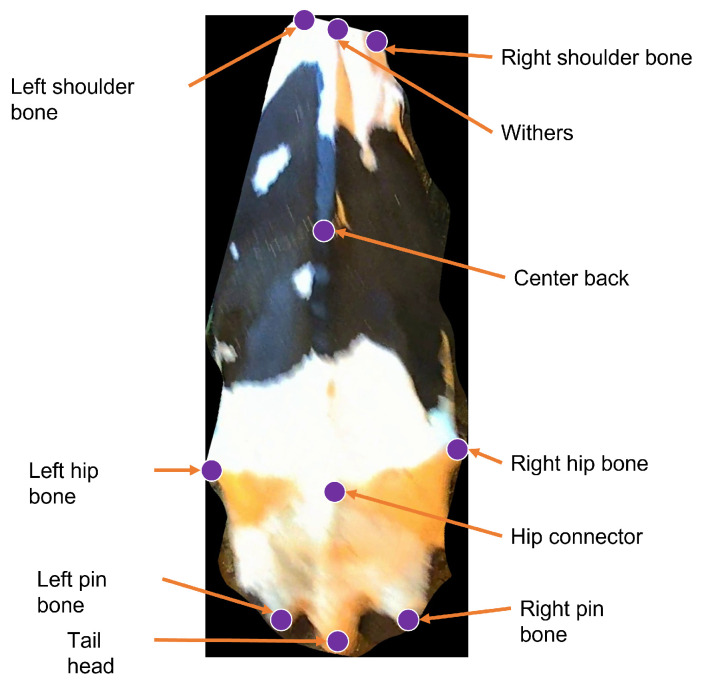
All ten keypoints labeled in top view. Image source: [1].

The eidetic cattle/cow recognition system, presented in [1], first extracts the ten keypoints illustrated in Figure 3 and an instance mask from the top view cow image using a keypoint-RCNN/mask-RCNN model [8]. The keypoint-RCNN/mask-RCNN model incorporates a Feature Pyramid Network (FPN) built on a ResNet101 (R101) backbone. Next, the system tries to interpolate missing keypoints with a keypoint interpolator that uses handcrafted interpolation policies. The system then eliminates any cow instance from further processing if the set of keypoints fails a set of twenty-one rules checked by a *rules checker*. This set of twenty-one rules enforces shape constraints by comparing the computed values of angles between keypoint triplets, distances between keypoint pairs, or ratios of such angles or distances to heuristically predetermined upper or lower bounds.

The semantic-instance mask is used to mask out the background, while the keypoints are used to align the cow image to a predefined template using affine transformations. The template-aligned image is then binarized and pixelated to obtain a QR code-like 2D data matrix, which is then serialized to form a bit vector. This bit vector is then compared with bit vectors in the cattlog database to find the nearest match. The cow ID associated with the matched bit vector is the predicted cow ID for that cow instance.

**Figure 4:**
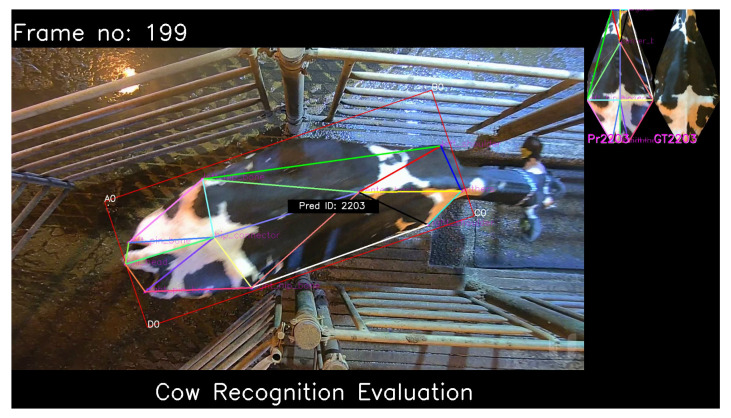
Our visualization tool with an example of the cow recognition system correctly predicting the cow ID for the cow in the given image from the holding area. The two images on the top right of the figure are template-aligned images—one on the left produced from the given image and the other on the right fetched from the cattlog (cattle catalog). The rotated bounding box computed from the instance mask, the keypoint skeleton, and the predicted cow ID (‘2203’) are all overlaid on top of the cow instance.

We process each video frame using this cow recognition system and obtain the predicted top-1 cow ID. An example is shown in Figure 4. We obtain these predictions from each video frame and use the most frequently predicted cow ID as the final, video-level prediction. The subsequent most frequent predictions form the top-K video-level predictions from the system. Note that to be considered for the video-level top-K predictions, a cow ID should be predicted with the highest confidence in at least one frame of the video.

In the context of eidetic cattle recognition, a *‘complete set’* is now the set of keypoints in which each of the ten detectable keypoints is marked, either directly by the keypoint detector or interpolated by our interpolator. This rules checker classifies whether the complete set of keypoints is erroneous or not.

### 3.4. Hard Instances of Cow Keypoints

In our context of cow identification, hard instances are cow instances with keypoints that are incorrectly produced by the detector model but are correctable by the keypoint rectifier. In the above definition, all keypoints in a complete set are declared to be in their correct locations if the set passes all rules of RulesChecker1 (RC1); otherwise, the set is said to have misplaced keypoints.

The cow identification accuracy depends on the system receiving cow keypoints in the correct locations. We also prefer training the keypoint detectors only on images where the cows are completely visible so that we can detect all keypoints and can obtain all blocks of its QR code-like data matrix to obtain a more accurate identity estimate. However, if the keypoint detector mistakenly detects a complete set of keypoints on a cow instance where all keypoints are not actually visible, that keypoint set is flagged by the rules checker and is prevented from being used for cow identification.

### 3.5. Implementation of the Cow Keypoint Rectifier

We now explain the working of the keypoint rectifier that is used to generate hard instances for self-training the cow keypoint rectifier. This rectifier also contributes to improving the cow identification accuracy by correcting misplaced keypoints during inference.

As described below, we use two different modes to correct the misplaced keypoints, and we reject the instance from being used for cow identification if both modes fail. At the crux of both these modes, the following steps are involved:

1. Run a rules checker on the complete set of keypoints and record the broken rules.
2. Determine the misplacement confidence for each keypoint from the list of broken rules using a reverse mapping.
3. Estimate which keypoints are misplaced from the misplacement confidence values using an estimator from a bank of estimators.
4. Delete all keypoints that were estimated to be misplaced.
5. Use the keypoint interpolator from [1] with a few new interpolation policies to interpolate all the deleted keypoints.
6. Use the new set of keypoints if they conform to all rules of RulesChecker1. Declare success and exit.
7. Go to (3) if there are untried estimators in the estimator bank. Otherwise, declare failure and exit.

The keypoint interpolator from [1] mentioned above plays an important role in the keypoint rectification procedure. It works by leveraging the redundancies in keypoint annotations. Redundancy here arises from the shape model of the keypoint skeleton—a few available keypoints could be used to estimate the position of one or more missing keypoints. For example, sections of the spine of the cow are used as lines of symmetry to estimate the location of one hip bone, pin bone, or shoulder given the other. A missing keypoint on the spine is interpolated by fitting a second-degree polynomial curve through the three available keypoints and then selecting a point on the curve that is at a certain computed distance from the three keypoints.

As mentioned earlier, we insert the keypoint rectification unit in the cow recognition system from [1] to obtain the updated cow recognition system as shown in Figure 1. The keypoints are checked for the 21 rules by the rules checker from [1]; failing which, keypoint rectification will be attempted.

We now explain keypoint rectification in detail. First, to create the reverse mapping from the list of broken rules to the misplacement confidence for each keypoint, we cannot use the rules checker from [1] (RulesChecker 1 (*RC1*)). This is because it has too few rules (21) for effectively resolving which keypoints are misplaced. Also, the limits for each of these rules are hand-crafted, making them not scalable. So, we create a new rules checker, which we call RulesChecker2, or *RC2*, with 124 rules whose limits are sourced from the ground truth data—the training set of the Cow Keypoints Dataset from [1], which has 717 images with annotated instances (also see Section 4). Since they are sourced from the human annotations, we call these the *Annotation Limits*. Note that half of these 124 rules check for compliance with the upper bound and the other half checks for compliance with the lower bound.

Note here that, to be consistent with the method used in [1], we still use RC1 to check for the correctness of the set of instance keypoints. That is, RC1 still has the authority to disallow an instance from reaching the cow ID prediction stage. RC2 is used only to help us estimate and correct the misplaced keypoints.

#### 3.5.1. The Reverse Mapping

After obtaining the list of broken rules from the new rules checker, the next step is to apply a reverse map to this list to compute misplacement confidence values for each keypoint.

The forward mapping is given by the rules checker, which maps the locations of keypoints to pass/fail values for each rule. The reverse mapping must help us estimate which keypoints are misplaced using the list of broken rules. To estimate which keypoints are misplaced, we compute and use the vector $\vec{C}=\left[c_{1},c_{2},\ldots ,c_{10}\right]$, where $c_{k}$ is the misplacement confidence value for keypoint k∈K, to rectify the keypoints. Here, *K* is the set of all 10 keypoints. The formula for computing $c_{k}$ is given in Equation (3).

(3) $$c_{k}=\frac{\#\mathrm{broken}\;\mathrm{rules}\;\mathrm{involving}\;\mathrm{the}\;\mathrm{keypoint}}{\mathrm{Total}\#\mathrm{rules}\;\mathrm{involving}\;\mathrm{that}\;\mathrm{keypoint}}$$

In essence, $c_{k}$ counts the number of ‘strikes’ or ‘hits’ received by a keypoint for breaking rules and then normalizes the total number of strikes by the number of rules the keypoint is associated with (because not all keypoints are associated with the same number of rules). This number $c_{k}$ is then used as the keypoint misplacement confidence. For instance, if eight rules involving the tail–head keypoint are broken and the tail–head keypoint appears in twenty rules, then the *c* value would be 0.40, implying the algorithm is 40% confident that the tail–head keypoint is misplaced.

Next, we sort the elements of $\vec{C}$ in descending order to obtain a sorted list of keypoint misplacement confidence values.

#### 3.5.2. Keypoint Rectification Strategy

After obtaining the sorted list of keypoint misplacement values $\vec{C}$, according to the core procedure described above, we use it to estimate and correct the misplaced keypoint. This estimation and correction is performed with the help of the two modes of keypoint rectification:

1. Iterative Mode;
2. Strategic Brute Force (SBF) Mode.

Before delving into the specifics of these two modes, we explain the scheme in which they will be used—the keypoint rectification strategy.

**Figure 5:**
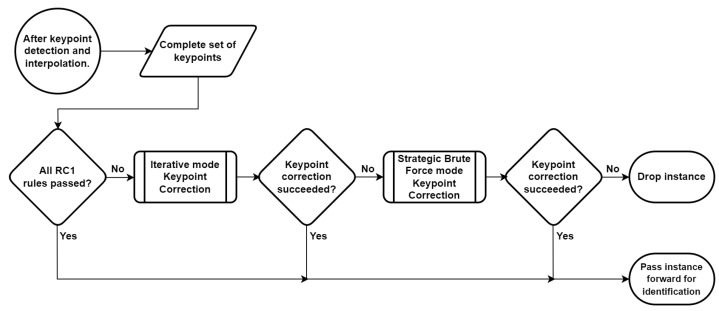
Keypoint rectification flowchart.

If the set of instance keypoints from the keypoint detector passes all the rules of RC1, we use the instance for identification. Otherwise, we try to estimate and correct the misplaced keypoints using the Iterative Mode. If Iterative Mode succeeds in rectifying the keypoints, we use the new set of keypoints and the cow instance for identification. Otherwise, we try to estimate and correct the misplaced keypoints using the SBF Mode. If the SBF Mode succeeds, we use the new set of SBF rectified keypoints and the instance for identification; otherwise, we preclude the instance from being used for identification. For either mode to be considered successful, we need the rectified set of keypoints to comply with both RC1 and RC2. We need all rules of RC2 to be passed so that the misplacement confidence value *c* for all keypoints is zero. We need all rules of RC1 to be passed for the results to comply with those of [1]. This strategy is illustrated in Figure 5.

We now explain the two modes of keypoint rectification in detail.

**Iterative Mode:** In the first approximation of this correction mode, we attempt to correct the keypoints one at a time until the set of keypoints passes all the rules from RC2. In each iteration, we obtain the sorted list of keypoint misplacement confidence values $\vec{C}$ as described above. We estimate the keypoint with the highest misplacement confidence value as the misplaced keypoint. We call this the ‘worst-hit keypoint’ as it has accumulated the most number of hits. We then delete this keypoint and interpolate a new one in its place. We continue iterating as long as the sum of misplacement confidence values $S=\sum_{k\in K}c_{k}$ is reducing. The reasoning behind this strategy is that once we correct a predicted misplaced keypoint, it no longer actively breaks rules, and its *c* value reduces. This allows the algorithm to estimate the next misplaced keypoint to be the one with the highest *c* value from the new set that now includes the corrected keypoint. Previously corrected keypoints can be used to correct/interpolate the next keypoints in need of correction. If the value of *S* remains the same across two consecutive iterations, the loop ends. If the final set of keypoints has an *S* value of 0 (all rules passed), we use this set as our corrected keypoints. Otherwise, we declare that this mode has failed.

However, this first approximation has a drawback. Recall that the cow recognition system in [1] interpolates keypoints that are missed by the keypoint detector using the positions of those that are detected. So, the problem with this approach would be that the worst-hit keypoint could be a keypoint that is already interpolated using locations of other wrongly predicted keypoints. This approach would then fail because it would delete the interpolated keypoint and interpolate another one exactly in its place. Then, the total number of hits (and hence *S*) would never decrease. Therefore, we need to modify this approach to handle multiple misplaced keypoints simultaneously.

A problem that manifests itself upon trying to correct the first two worst-hit keypoints simultaneously in every iteration is that the second worst-hit keypoint might not actually be misplaced. It can just so happen that the keypoint with the highest *c* value shares many rules in common with a correctly detected keypoint, and some of these rules are broken. Since these broken rules are associated with both these keypoints, our algorithm assigns one strike to both of them for every such broken rule. The correctly detected keypoint could then accumulate enough strikes to make it to the second spot in the sorted list $\vec{C}$, leading to a failure if we tried to correct it.

Nonetheless, by virtue of how the rules were designed, a pattern emerges: when one hip bone is the worst-hit keypoint and the other hip bone is the second worst-hit, both the hip bones are actually misplaced. The case is the same with pin bones. We call such keypoint pairs ‘keypoint mirrors’. In a similar fashion, it is often the case that if the center back is worst-hit and the hip connector is second worst-hit, both of them are actually misplaced. We call such keypoint pairs ‘accomplices’. This list includes (hip bones, hip connector) and (tail head, hip connector) pairs. When such cases occur, we declare both keypoints to be misplaced. We also added a rule based on heuristics to include the tail head in the list of predicted misplaced keypoints if certain rules were broken. This is because we found that the misplaced-keypoint-estimation algorithm tends to ignore it.

We therefore modify our first approximation algorithm to delete and interpolate anew all keypoints that are predicted to be misplaced—worst-hit keypoint, along with mirrors, accomplices, and tail–head keypoints as required, in every iteration. If the corrected set of keypoints passes all rules of RC2, we further subject it to RC1 and declare success only if all rules of RC1 are also passed. This is to ensure that the instances that reach the cow ID prediction stage in the cow recognition system remain consistent with that of [1].

**Figure 6:**
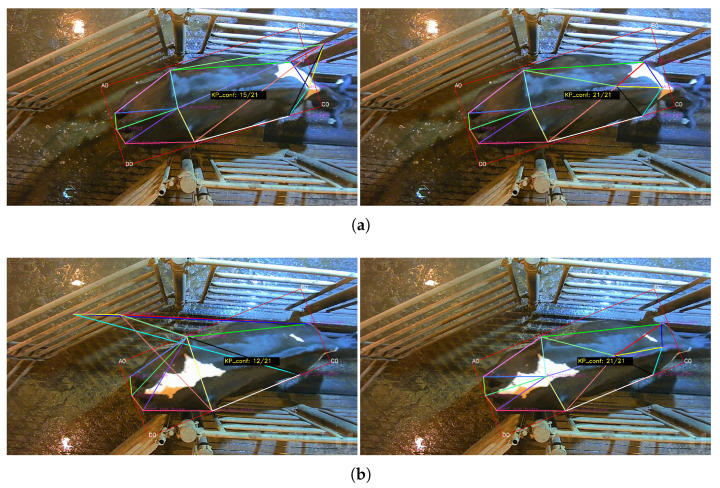
Some examples of keypoint predictions before and after successful keypoint rectification using the Iterative Mode. The number of rules passed out of the 21 rules of RulesChecker1 is overlaid as ‘KP_conf’. (**a**) Example where only one keypoint (center–back) is corrected by placing it on a second-order polynomial curve fit through the spine points that are correctly detected. (**b**) Case that Iterative Mode was designed to handle—first, the hip connector is corrected, then the withers, and then the two corrected keypoints together with the tail–head keypoint are used to interpolate/correct the misplaced center–back.

Examples of the Iterative Mode of keypoint rectification can be found in Figure 6a,b.

**Strategic Brute Force (SBF) mode:** Sometimes, because multiple keypoints are affected by the same rule, the highest *c* value does not correspond to the misplaced keypoint. The misplaced keypoint could be any other keypoint that has accumulated at least one strike. We also see cases where there are more than one misplaced keypoint, none of which have accumulated the most strikes. An example of this could be if both hip bones are misplaced. Since hip bones share many rules in common with the hip-connector keypoint, the hip connector would accumulate strikes from these common rules and, in turn, attain the highest *c* value.

**Figure 7:**
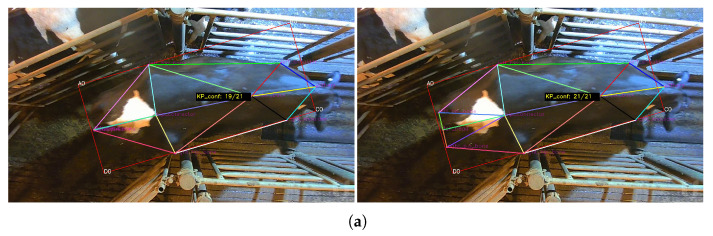

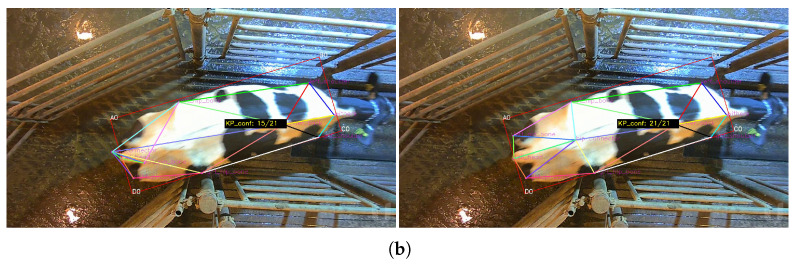
Some examples of keypoint predictions before and after successful keypoint rectification using the SBF Mode. The number of rules passed out of the 21 rules of RulesChecker1 is overlaid as ‘KP_conf’. (**a**) Case where both the pin bones are misplaced. The worst-hit keypoint will usually be the tail–head as it is involved in broken rules associated with both pin bones. SBF Mode removes the second and third worst-hit keypoints, which are the two pin bones, and interpolates new ones in their places. (**b**) Case where SBF Mode removes the left hip bone and the hip-connector keypoints and corrects them. This is a case where Iterative Mode fails, going against expectation, and SBF Mode comes to aid.

Examples of SBF Mode of keypoint correction can be found in Figure 7a,b.

To deal with such cases, we apply a brute-force approach to correct the keypoints using the same basic procedure—estimate misplaced keypoints, delete them, interpolate new ones in their places, and check if the new set of keypoints breaks no rules from RC2. We optimize this brute-force approach by estimating only those keypoints that have a non-zero *c* value to be misplaced. If this fails, we estimate more than one keypoint to be misplaced simultaneously—the keypoints with the three largest *c* values, in combinations of two and then all three at once. Since this has more logic and heuristics involved than naive brute-forcing, we call it the Strategic Brute Force mode.

In each of these correction attempts, if the new set of keypoints has all zero *c* values (i.e., all RC2 rules passed), we subject the set to RC1 as well, as mentioned earlier, to maintain consistency in the quality of detected keypoints with that of [1]. If the set of keypoints passes all rules from RC1, we declare the mode to be successful and proceed with this set of corrected keypoints. Otherwise we declare that this mode has failed.

## 4. Datasets

In this section, we present the datasets on which we perform the experiments described in Section 6. We begin with a quick overview of how we collected and curated data to create these datasets and then describe each dataset in detail.

In Section 6, we describe our experiments, which include half-day experiments to explore SURABHI and its role in improving cow recognition and whole-day experiments to enable a direct comparison with the results of [1]. The half-day experiments use datasets that are much smaller than those of whole-day experiments. Next, we explain the datasets used for whole-day experiments and follow it with the explanation of how we split the data into smaller datasets for half-day experiments. We then detail the classification of the data used to train the keypoint detectors for both half-day and whole-day experiments. Finally, we explain how the sets of easy and hard instances are sampled and combined with the human-annotated instances to create the datasets used for self-training models for each experiment presented in Section 6.

### 4.1. Overview of the Data

We use the same set of raw videos from [1] for the experiments in this paper, which are the videos we recorded at the Purdue Dairy from the top view, of cows walking one at a time in the holding area in Summer 2021 and two days of Summer 2022 (Day1 and Day2), along with videos of cows in the barn in Summer 2022. The resolution of all these videos is 1920 × 1080 px (FHD). The holding area is the area where only one cow is allowed to walk across the scene at a time, and the barn is an unconstrained area where multiple cows can be present in the scene simultaneously. The raw videos from the holding area from Summer 2022 are cut into segments with only one cow walking across the scene. We call these the cut-videos.

When we refer to individual datasets such as Day1 or its subsets, we mean the dataset of the cut-videos by default. We use the extension .cows to refer to the set of individual cows whose cut-videos are contained in a dataset. Individual cows are referred to by their four-digit cow IDs (e.g., 4455). When we say that a cow is in a dataset (say Day1), we mean that the cut-video of the cow is in the dataset. This automatically implies that the cow ID is present in the set dataset.cows.

Day1 has 153 cows. Day2 has 170 cows, of which 148 are also in Day1. In [1], this set of 148 common cows was referred to as the Test-InSet. In this paper, we ignore the 22 additional cows from Day2 and use the name Day2 to refer to this set of 148 common cows.

As before [1], we use images of cows along with annotated keypoints and instance masks to train the keypoint and mask detector deep-learning models. Since we are concerned with the end-to-end performance of the updated cow recognition system (Figure 1), instead of focusing on the evaluation results of the keypoint/mask detection models independently, we use the cow identification accuracy of the entire system as an indirect performance metric. So, we evaluate the performance of the updated cow recognition system directly on cut-videos.

In these datasets, an instance refers to a cow in an image (a video frame), together with its keypoints and segmentation mask (instance mask) annotations. Since each cut-video has only one cow in it, we sometimes use the term ‘instance’ to also refer to the image containing the cow instance. So, when we say that *n* instances are added to the training dataset of the keypoint/mask detector, we mean that the images containing the single cow instances are added to the training dataset.

### 4.2. Datasets for Whole-Day Experiments

For whole-day experiments, similar to [1], we train the keypoint detector on images of all cows that appear on the whole day of Day1, and also on images of cows in the barn area. For these experiments, all additional training instances for SURABHI are sourced from Day1. Again, similar to [1], the system containing these models is evaluated on cut-videos of the 148 from Day2.

### 4.3. Splitting the Datasets for Half-Day Experiments

In a typical dairy setting, new cows are added and removed on a regular basis. We need the stochastic, learning-based, keypoint detector to perform well on both seen and unseen cows. So, for the purpose of evaluating performance on both seen and unseen cows in whole-day experiments, we divide the sets of cows on both days into two smaller subsets, each with roughly the same number of cows. We randomly partition cows on Day1 into two sets: Day1-SetA with 76 cows and Day1-SetB initially with the remaining 77 cows. From Day2, we create SetA with 73 cows and SetB with 75 cows, not by random partitioning, but in such a way that these two sets of cows are subsets of the corresponding ones on Day1. Further, to enable a comparison of the average performance of the different models on unseen cows from both days of Summer 2022, we desire to have the same cows in SetBs of both days. These set relationships are represented below:

(4) $$\begin{array}{c} Day2SetA.cows\subseteq Day1SetA.cows; \end{array}$$

(5) $$\begin{array}{c} Day2SetB.cows=Day1SetB.cows. \end{array}$$

**Figure 8:**
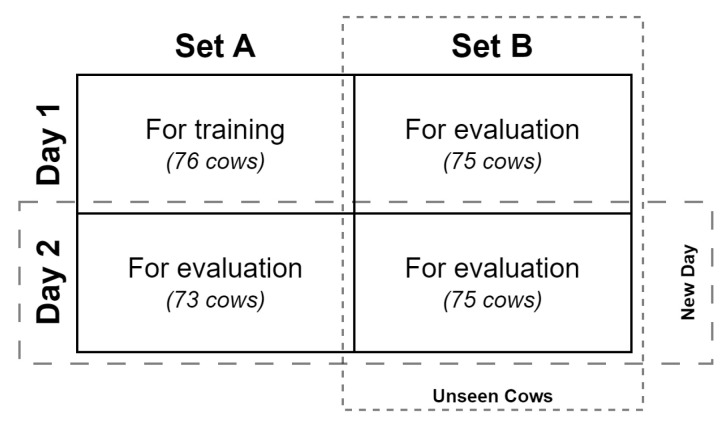
Evaluation strategy for whole-day experiments: The cut-videos from Summer22 Day1 and Day2 are split into SetA and SetB each. Day1-SetA videos are used for training the keypoint detector model. The evaluation results from the three other subsets are combined to obtain new insights as shown.

To satisfy Equation (5), we remove from consideration two cows from Day1-SetB that are not present in Day2-SetB. This brings the total number of cows in Day1-SetB down to 75 from the previous 77 (see Figure 8).

For whole-day experiments, we use images of all cows in the Day1-SetA for training the models and directly evaluate on the cut-videos of all cows in the other three subsets (Day1-SetB, Day2-SetA, and Day2-SetB). We use the strategy detailed in Section 5 to combine the evaluation results from these three subsets to gain deeper insights.

### 4.4. Classification of Training Data for Keypoint Detection

We now describe how the training data for the keypoint detector are classified to form different subsets. The different half-day experiments explore the performance of keypoint detectors when trained on these data subsets. The same methodology is then carried forward to whole-day experiments.

**Figure 9:**
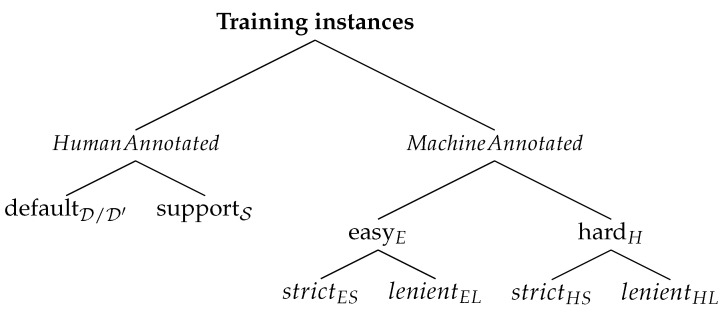
Classification of training data. Each node is a set of instances. The subsets at children nodes of MachineAnnotated dataset do not necessarily partition the sets at their parent nodes. The strict sets are in fact subsets of the sibling lenient nodes. We show them as siblings instead of showing a parent-child relationship as we are explaining only the classification.

The trees in Figure 9 illustrate how annotated instances are classified under different sub-sets. All these annotated instances are of cows designated for model training in the respective experiments—Day1-SetA for half-day experiments and Day1 for whole-day experiments. We train the models on different combinations of these data subsets in the following experiments. Although annotations include both keypoints and instance masks, when we refer to annotated instances in the explanation that follows, we are concerned mainly with the keypoint annotations. This is because the instance mask detection performance is already very good [1].

The main branches in the trees in Figure 9 are as follows:

1. HumanAnnotated, which is the subset that contains instances whose ground truth labels come from human annotators.
2. MachineAnnotated, which is the subset that contains instances whose ground truth labels come from the keypoint detector-interpolator output or from the keypoint rectifier output of the updated cow recognition system (Figure 1).

This default subset of the HumanAnnotated dataset is referred to by $D^{\prime }$ for half-day experiments and D for whole-day experiments. This subset contains just a single annotated instance per training cow in full view. The difference in notation is to maintain a distinction between the larger default dataset for whole-day experiments (D), which contains one annotated instance for each cow in Day1, and the smaller default datasets for half-day experiments ($D^{\prime }$), which contain one annotated instance for each cow in the smaller Day1-SetA dataset.

The ‘support set’ or S dataset contains human-annotated images from the Summer21 holding area and Summer22 barn area. There can be more than one image of the same cow in this set. This set will not be used for half-day experiments and will only be used for whole-day experiments.

Machine-annotated instances are generated by first training the keypoint detector models on annotated images in a subset of the HumanAnnotated dataset as the seed dataset and then using these trained models to infer on the cut-videos of training set cows of the respective experiments. Machine-annotated instances in the subset (MachineAnnotated) are classified as ‘easy’ if the annotations are directly from the keypoint detection model and all keypoint rules are passed. Note that these instances are considered ‘easy’ even if some of the keypoints are interpolated by the keypoint interpolator.

Machine-annotated instances are classified as ‘hard’ if the keypoint annotations pass all the rules (of both rule checkers) only after correction by the keypoint rectification sub-module. These subsets can contain more than one annotated image per cow depending on how the keypoint detectors perform on cut-videos of their training datasets.

In the trees of Figure 9, HS and HL stand for Hard&Strict, and Hard&Lenient datasets. Similarly, ES and EL denote Easy&Strict, and Easy&Lenient datasets. The need and procedure for creating these datasets are explained next in Section 4.5.

### 4.5. Creating the Training Datasets for Experiments

In this section, we explain how the different sets of data in Figure 9 are combined to create the training datasets for each experiment. For both half-day and whole-day experiments, we first train a baseline model each and use the system with this model to generate the baseline results. Later, we apply this baseline system to cut-videos meant for training to produce the additional hard and easy instances required for the experiments.

We first describe the datasets used to train the baseline models. Next, we explain how we create the datasets with additional hard and easy instances.

#### 4.5.1. Datasets for Generating the Baseline Results

For half-day experiments, we use $D^{\prime }$ with 76 images—one per cow in Day1-SetA, as the training dataset. In whole-day experiments, the default human-annotated dataset (now D) contains one image per cow for every cow in Day1. We unite this dataset D with the support set S (see Figure 9) to form the HumanAnnotated dataset:

(6) HumanAnnotated=D∪S.

This resulting HumanAnnotated dataset is the same as the training set of the Cow Keypoints Dataset in [1], and is used to train the baseline models in whole-day experiments.

#### 4.5.2. Datasets with Hard Instances

To generate the hard instances, we first apply the baseline systems to the cut-videos sequestered for sourcing the training instances. These are the cut-videos of Day1-SetA for half-day and Day1 for whole-day experiments. We then collect the keypoint-rectified instances as hard instances.

To isolate high-quality training instances to ensure model robustness, we filter all hard instances obtained above using the two conditions listed below:

- C1: The list of corrected keypoints must not include either shoulder.
  - Reason: the shoulder region of the cow is most susceptible to movement, and hence to correction errors.
- C2: The spine of the cow must be straight. We check this by first fitting a second-order polynomial curve through the visible spine points and then limiting the value of the coefficient of the second-order term.
  - Reason: In our keypoint correction scheme, misplaced edge keypoints are corrected by reflecting the corresponding ones on the opposite side of the cow across the spine. A bent spine could result in the reflected keypoint being where it should not be.

We call this filtered set of hard instances the lenient set, and it is denoted by HL for Hard&Lenient. Further, we filter instances from the lenient set as per condition C3 below.

- C3: the cow instances must be correctly identified.
  - Reason: A miscorrected set of keypoints could have led to an incorrect identification for that instance. We need to eliminate such possibilities.

The resulting subset of hard instances is called the strict subset and is denoted by HS for Hard&Strict. This classification is shown in Figure 9.

Remember that we filter and classify hard instances into strict and lenient only for training the keypoint detectors and not when evaluating the system. This holds for both half-day and whole-day experiments. Also note that the HS and HL datasets for half-day experiments have instances of cows only from Day1-SetA but could have instances of any cow from Day1 for whole-day experiments.

These sets of hard instances are then augmented with human-annotated datasets to create two expanded (*X*) datasets—HSX and HLX—which are used to train the models. For half-day experiments, the expanded datasets $HSX^{\prime }$ and $HLX^{\prime }$ are generated using the datasets in Figure 9 according to the equations below:

(7) $$\begin{array}{c} HSX^{\prime }=HS\cup D^{\prime }; \end{array}$$

(8) $$\begin{array}{c} HLX^{\prime }=HL\cup D^{\prime }. \end{array}$$

For whole-day experiments, the expanded (*X*) datasets are created by uniting the hard instance sets with the larger HumanAnnotated dataset instead of just the default dataset *D*:

(9) $$\begin{array}{cc} HSX & =HS\cup HumanAnnotated; \end{array}$$

(10) $$\begin{array}{cc} HLX & =HL\cup HumanAnnotated. \end{array}$$

#### 4.5.3. Datasets with Easy Instances

To generate easy instances, we apply the baseline systems for the respective experiments to the cut-videos set aside for training. Again, these are cut-videos from Day1-SetA for half-day and Day1 for whole-day experiments. We then collect instances that are correctly localized (pass all rules of RC1) without keypoint rectification as easy instances.

This set of all easy instances is called the lenient set and is denoted by ES for Easy&Lenient. Similar to what we did with hard instances in Section 4.5.2, we use condition C3 to create a strict subset by including only those easy instances that resulted in correct cow identifications. This set is denoted by ES for Easy&Strict. The ES and EL datasets for half-day experiments have instances of cows only from Day1-SetA, but for whole-day experiments, they could have instances of any cow from Day1.

We then augment these two datasets with the default human-annotated datasets to produce two expanded (*X*) datasets—ESX and ELX—which are used to train the models. For half-day experiments, the expanded datasets $ESX^{\prime }$ and $ELX^{\prime }$ are given by the equations below:

(11) $$\begin{array}{cc} ESX^{\prime } & =ES\cup D^{\prime }; \end{array}$$

(12) $$\begin{array}{cc} ELX^{\prime } & =EL\cup D^{\prime }. \end{array}$$

Again, for whole-day experiments, the expanded (*X*) datasets are created by uniting the hard instance sets with the larger HumanAnnotated dataset instead of just the default dataset *D*:

(13) $$\begin{array}{cc} ESX & =ES\cup HumanAnnotated; \end{array}$$

(14) $$\begin{array}{cc} ELX & =EL\cup HumanAnnotated. \end{array}$$

## 5. Evaluation Criteria, Strategy, and Metrics

In this section, we first define the criteria for asserting an improved keypoint detector. We then discuss the primary evaluation metrics that we use to measure the performance of the keypoint detector in whole-day experiments. These include metrics that measure the end-stage identification accuracy, as well as one that measures the keypoint detection performance using a rules checker.

Since whole-day experiments compare self-trained models from this paper with the one in our previous work [1], which used the primary evaluation metrics, these metrics are sufficient for the task. However, since the half-day experiments explore the proposed method SURABHI, they need a different evaluation strategy and evaluation metrics that support this strategy. So, next we discuss this evaluation strategy for measuring the performance of the keypoint detector in half-day experiments. Then, we explain additional evaluation metrics derived from the primary metrics to support this strategy.

### 5.1. Evaluation Criteria

We consider a keypoint detector to be better if it improves our cow-ID prediction accuracy. Therefore, we measure the performance of the keypoint detector indirectly by evaluating the entire cow recognition system end-to-end. Moreover, a training strategy is better when it improves keypoint detection on new cows or in new environments. Here, new cows are defined as those not seen by the keypoint detector while training. A new environment is any setting different than in the training data; for example, the same cow seen on a different day in the same location corresponds to a new environment due to changes in scene background, scene lighting, and in the location of the cows.

### 5.2. Primary Evaluation Metrics

As mentioned before, we use the cow identification accuracy as a means to measure the performance of the keypoint detector model. Additionally, we have a metric that measures the keypoint detection performance using a rules checker. We use the evaluation dataset to evaluate the cow recognition system both at the video level and at the instance level. At the video level, we measure the *top-K video-level accuracy*. At the instance level, we measure the *number of correct identifications* and the *number of correct localizations*. All the primary evaluation metrics discussed below are common to both half-day and whole-day experiments. An increase in any of these metrics indicates a better keypoint detector.

- **Top-K video-level accuracy:** Top-K video-level accuracy is computed from top-1 frame-level results. Specifically, top-K predictions at the video level are compiled by collecting the top-1 cow ID predictions for each video frame and sorting these predicted cow IDs in decreasing order of frequency of occurrence. If the correct prediction is in the top K, then the cow ID prediction is said to be top-K accurate. The maximum meaningful value of K is the maximum number of different identities a system obtains for any cow in the dataset. In particular, increasing the value of K beyond this maximum value will not increase video-level accuracy. We report the Top-Max accuracy as the accuracy for the maximum meaningful value of K.
- **Number of correct identifications:** This number gives the total number of cow instances, across all frames in all cut-videos of the evaluation dataset, for which the predicted cow ID is the same as the ground truth cow ID. This enables us to probe the system deeper, at the instance level, for detecting improvements that can be missed by the top-K video-level accuracy.
- **Number of correct localizations:** This quantifies the number of cow instances, across all frames in all cut-videos of the evaluation dataset, for which the final set of keypoints passes all rules of RuleChecker1 (RC1). This final set of keypoints includes those that are directly detected by the keypoint detector, those that are missed by the keypoint detector but interpolated by our interpolator, and even those rectified keypoints obtained from the keypoint rectifier. Remember that if the interpolator fails to interpolate all the missing keypoints, the number of rules passed is set to zero, and such instances are barred from being used for identification. Also, instances with rectified keypoints are only allowed to reach the cow ID prediction stage if all RC1 rules are passed.

Note that the metric ‘number of correct identifications’ depends on factors beyond the performance of the keypoint detector. For example, the performance of the binarization block (Figure 1) can be affected by illumination conditions, which would then decrease the ‘number of correct identifications’. So, we use the number of correct localizations to independently measure the performance of the keypoint detector model. Also, while the number of correctly identified instances can measure performance improvements, the top-K video-level accuracy (and the metrics derived from it below) shows whether the improvement is concentrated around a small set of individuals or if previously unidentified individuals are newly identified.

### 5.3. Evaluation Strategy for Half-Day Experiments

For measuring the performance of the keypoint detection models in half-day experiments as per the above evaluation criteria, we use the following strategy of combining the results from the individual datasets to obtain more insight:

- *Seen and unseen cows on a fresh day:* By summing the evaluation results from Day2-SetA and SetB, we obtain the total results of both seen and unseen cows on a dataset collected on an entirely different day (Day2) from what was used for training (Day1). This helps us measure the general performance of the keypoint detector models on both seen and unseen cows in an unseen background environment.
- *Unseen cows on distinct days:* Since the keypoint detector models are trained on Day1-SetA, all cows in SetB are unseen cows. Given that the cows in SetB from both days of Summer22 are the same (Equation (5)), we obtain the average performance of the keypoint detector model on two distinct days by averaging the performance metrics from the Day1-SetB and Day2-SetB datasets. This helps us measure the general performance of the models on unseen cows across different background environments from different days.

This strategy of combining evaluation metrics obtained from split datasets is illustrated in Figure 8.

### 5.4. Derived Evaluation Metrics for Half-Day Experiments

To support our evaluation strategy for half-day experiments, we derive the following metrics from the primary evaluation metrics. These derived evaluation metrics include those that measure the keypoint detection performance on all cows on a fresh day and also those that measure the performance on unseen cows on two different days.

#### 5.4.1. For Evaluating on Seen and Unseen Cows on a Fresh Day

For measuring the performance of keypoint detector models on seen and unseen cows (all cows) on a fresh day (Day2), we use the derived metrics listed below. An increase in any of these metrics indicates a better keypoint detector:

- **Total number of correct localizations:** This value is *the number of correct localizations* when the model is evaluated on the entire Day2 dataset (see Figure 8).
- **Total number of correct identifications:** This value is *the number of correct identifications* when the model is evaluated on the entire Day2 dataset (see Figure 8).
- **Combined top-max accuracy (%):** This metric refers to the maximum available *top-K video-level accuracy* value from evaluating the model on all cows on the new day, i.e., all cows in the Day2 dataset. This metric gives the percentage of cows that the system correctly identifies in at least one frame of their cut-videos. An increased value on this metric indicates an increased recall value, i.e., the system has identified more cows than before.

#### 5.4.2. For Evaluating on Unseen Cows on Distinct Days

For measuring the performance of keypoint detector models on unseen cows (from SetB) on two different days, we use the derived metrics listed below. An increase in any of these metrics indicates a better keypoint detector:

- **Average number of correct localizations:** This value is obtained by averaging the *the number of correct localizations* metric from Day1-SetB and Day2-SetB datasets. This number is rounded off to the nearest integer value.
- **Average number of correct identifications:** This value is obtained by averaging the *number of correct identifications* metric from Day1-SetB and Day2-SetB datasets. This number is rounded off to the nearest integer value.
- **Average Top-Max accuracy (%):** This metric refers to the maximum available *top-K video-level accuracy* value from evaluating the model on the combined set of cut-videos of unseen cows from both the days (see Figure 8). This metric averages out the identification accuracy for each cow because the system is made to identify the same cows on two different days. This metric gives the percentage of cows that the system correctly identifies in at least one frame of their cut-videos, averaged over two different days. An increased value on this metric indicates an increased recall value, i.e., the system has identified more cows than before.

## 6. Experiments and Results

In this section, we investigate the potency of SURABHI by applying it to the task of cow-keypoint detection. To test the effectiveness of SURABHI with the updated cow recognition system shown in Figure 1, we first run experiments on smaller, half-day datasets in Section 6.1. We show how training models on a dataset with additional hard instances is better than training them on a dataset with additional easy instances. In these experiments, our focus is on proving that SURABHI is effective and not on comparing the results from using strict and lenient hard instances. We then rerun the same experiments on the larger, whole-day datasets in Section 6.2 to compare the performance of the earlier approach in [1] with the new approach that uses keypoint rectification and SURABHI.

In all the experiments, we use images to train the models (keypoint/mask detectors) and videos to evaluate the updated system. As stated earlier, the performance of the overall system is used as a proxy for the performance of the keypoint detection model. The following experiments differ only in the set of images that are used for training the keypoint and mask detector block (model) and the set of videos that are used for evaluating the entire system. All blocks in the system remain the same for all the experiments, except for re-computing the EI2023 baseline in Section 6.2, for which we use the older system from [1]. That is, the system we use in all the experiments uses the keypoint rectifier when inferring a cow identity, except when recomputing the EI2023 baseline in Section 6.2. This is regardless of whether the models were retrained with SURABHI or not. The details of model training are in Appendix A.

In the following explanation, ‘model’ refers to a keypoint-RCNN/mask-RCNN deep-learning model that is built with a ResNet101-FPN backbone. The same term in plural, i.e., ‘models’, refers to the same keypoint/mask detector model trained on different datasets. For example, when we say that self-trained models are better than those that are not, we do not mean that the architecture of the models is different. Instead, we mean that the same model, when self-trained, performs better.

### 6.1. Half-Day Experiments

The half-day experiments evaluate the effectiveness of SURABHI using the half-day datasets, which allows us to explore both the impact of unseen cows and a new environment (a fresh day). In this section, we first briefly describe the experiments and then present a detailed discussion consolidating results obtained in all the half-day experiments. As described in Section 4.3, all models in half-day experiments are evaluated on the three sets of evaluation cut-videos. The results from each of these sets are combined as per the evaluation strategy (Section 5.3) and measured using the derived evaluation metrics (Section 5.4).

We first explore the performance of the models trained without our self-training technique, SURABHI, to provide a baseline for half-day experiments. For this, we train the keypoint detector model on the default human-annotated dataset $D^{\prime }$, use this trained model in the updated cow recognition system (Figure 1), and then evaluate the overall system. We refer to these evaluation results as the *Rectified Keypoint Baseline (RKB)*, as they are from the new system that uses rectified keypoints for cow identification.

Next, we compare the baseline results with the results from using models self-trained on additional hard and easy instances. For this, we first train models on the $HSX^{\prime }$, $HLX^{\prime }$, $ESX^{\prime }$, and $ELX^{\prime }$ datasets as described in Section 4.5 and then evaluate them.

**Figure 10:**
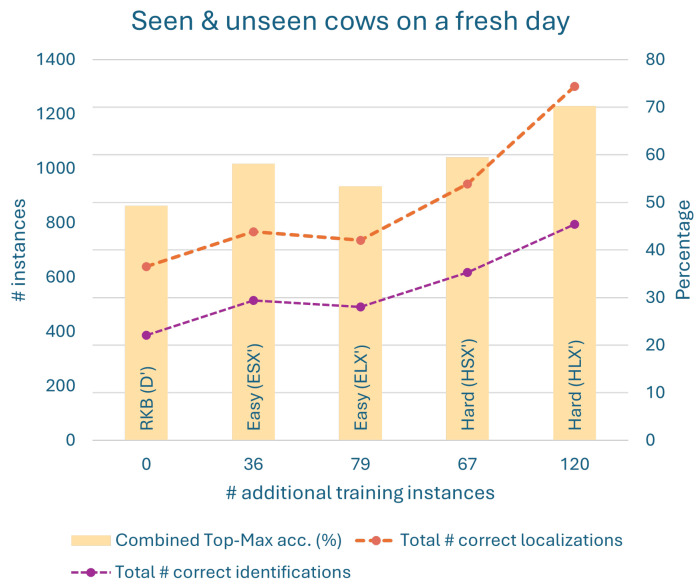
Plot of results from half-day experiments: evaluation on seen and unseen cows on a fresh day.

**Figure 11:**
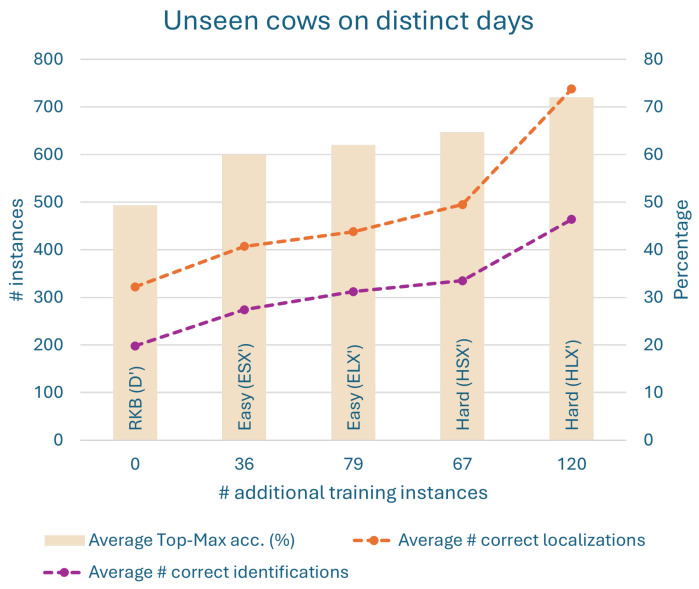
Plot of results from half-day experiments: evaluation on unseen cows on distinct days.

The results from all the above models are plotted in Figure 10 and Figure 11 using the derived evaluation metrics. These plots enable a detailed discussion of the results below in Section 6.1. Interested readers can find the same results presented in a table in Appendix B.

#### Discussion on Results of Half-Day Experiments

We now discuss the results of half-day experiments from the plots in Figure 10 and Figure 11. First, note the three factors that influence the results: the number of training instances, the lenience in selecting the training instances, and finally the hardness of those instances. We discuss the results with respect to these factors. Also observe that increasing lenience increases the number of training instances. This is because the set of strict instances is a subset of the set of lenient instances.

The keypoint-RCNN model does not perform well by default, but it always improves when more training instances are provided. For seen and unseen cows on a new day in Figure 10, an increase in selection lenience or in the number of training instances does not imply an improvement in performance (in any metric). This is because the results improve with additional lenient hard instances, while they deteriorate with additional lenient easy instances. Therefore, the performance improvement is because of the hardness of the training instances. For unseen cows on both days, Figure 11, the model trained on a stricter and smaller set of hard instances ($HSX^{\prime }$) outperforms the one trained on a lenient and larger set of easy instances ($ELX^{\prime }$) in all the metrics. This implies that hard instances are more effective and efficient.

Also, when working with easy instances, we have to make do with however many we have. If the default model is not good enough to produce many easy instances to train on, we just train on as many as we can obtain. There is no other way to produce more easy instances.

To summarize, it is clear from the results that SURABHI is indeed effective.

### 6.2. Whole-Day Experiments

The default models considered in half-day experiments are part of the updated cow recognition system (Figure 1). But the updated system itself is an upgrade over our previous system in [1]. We now test the effectiveness of both keypoint rectification and SURABHI in improving the results obtained in [1] by experimenting on the whole-day datasets. Models trained on the different datasets in these experiments are evaluated on Day2 cut-videos.

To compare results from our previous work [1], we generate the EI2023 baseline by training the keypoint detection models on the HumanAnnotated dataset as per Section 4.5.1, using these models in the older EI2023 cow recognition system from [1], and then evaluating them.

Next, similar to half-day experiments, we generate the Rectified Keypoints Baseline (RKB) results. For this, we first train the model on the HumanAnnotated dataset as explained in Section 4.5.1. This model is therefore the same as the one used in creating the EI2023 baseline. We then use this model in the updated cow recognition system with the keypoint rectifier (Figure 1) and then evaluate it.

We then perform experiments described in half-day experiments, but now on the whole day datasets. That is, we train models using the HSX, HLX, ESX, and ELX datasets for whole-day experiments as per Section 4.5 and evaluate them on cut-videos from Day2.

The training dataset information along with the evaluation results are in Table 1. As can be seen in the table, the new RKB outperforms the EI2023 baseline on all metrics. There are many more easy instances generated than hard instances, and yet, training models on these many easy instances proves to be detrimental. We find that the best performance on all evaluation metrics is achieved when the keypoint-RCNN model is trained on the HSX dataset. Further, it achieves this result with the fewest additional training instances. The strictness in selecting hard instances has proved to be an advantage in whole-day experiments. So again, SURABHI is effective.

Further, we include in Table 1 the results from an ablation study that deactivates the keypoint rectifier during evaluation. Without the keypoint rectifier during inference, the recognition system in this study is the same as the older system from [1]. From the results of this study, we see that models trained on hard instances outperform the comparison baseline (EI2023 Baseline [1]). Additionally, the model trained on the HSX dataset outperforms even RKB, implying that a model trained with SURABHI is better than simply including a keypoint rectifier. Of course, the system performs best when both of them are used together—SURABHI to train models and the keypoint rectifier to assist localization during inference.

To summarize the results, application of SURABHI helps us correctly identify cows in 414 (22%) more instances while also identifying two additional cows, on top of what the RKB achieves.

## 7. Conclusions

We develop SURABHI, a self-training method that reinforces a keypoint detection model using hard instances, and use this method to improve the performance of a top-view cow-keypoint detector. To aid this, we develop a system that uses shape priors to detect and rectify misplaced keypoints on cow instances and collect such machine-rectified sets of keypoints as ‘hard instances’. We then augment the training dataset of the keypoint detector with some of these hard instances to reinforce the keypoint detector model exactly in those places where it is lacking. We show that the performance of the keypoint detector improves after it is trained on such datasets.

SURABHI proves to be very effective in training models with high capacity when the available seed training data are so minimal that they are not only insufficient to produce a good-performing model but also cannot produce enough instances with correct keypoint detections for improvement with self-training. We then show that using a large number of instances with correct keypoint detections is in fact detrimental to the performance of the model and that the quality of instances used for self-training matters. Hard instances guarantee this quality. On direct comparison with our previous work [1], we find that our updated cow recognition system, when combined with SURABHI, outperforms the cow recognition system in [1] by a huge margin.

Although we cannot guarantee a performance improvement in all cases, since SURABHI is agnostic to the underlying detection models, it can be applied to any keypoint detector model to seek improvement. In this paper, we illustrate a case where SURABHI does improve the performance of the keypoint detector and of the downstream vision task.

### 7.1. Limitations

The rules checkers we use are the only guardrails against propagating keypoint detection errors. So, the rules checkers are designed to be stringent to not miss any keypoint errors. Therefore, any miscorrected keypoint instance is caught and is prevented from being used for identification. However, the rules checker is sometimes too strict and does not allow correctly rectified keypoint sets to pass. This over-strictness is because the rules checker cannot learn to account for all possible cow poses from the small training dataset. Although there might be a few training instances lost due to this strict rules checker, the results with SURABHI are still very good.

### 7.2. Future Work

Future work could include developing a system that automatically learns the constraints and relations in keypoint locations and generates the most optimum set of rules for estimating keypoint detection errors. This would allow us to create a generalizable model that can learn the shapes of any deformable object. Such a shape model should allow application of keypoint rectification, and thus SURABHI, readily to objects of any shape.

## Figures and Tables

**Table 1:** Results for whole-day experiments. The results in this table use the primary evaluation metrics. The best model is the one with the highest number of correct localizations, identifications, and video-level top-K accuracy for the highest possible K value. The highest values in each evaluation column are highlighted in bold.

	Training Dataset Information			Evaluation Information					
	Day1					* Video Level Top-K Accuracy *			
Experiment	Dataset	#Training	#Easy/Hard	#Correct	#Correct	* (of 148 Cows, in %) *			
Info	Name	Images	Inst. Used	Localizations	Identifications	1	2	4 (K<4)	9 (K<9)
EI2023 Baseline [1]	Human Annotated	893	N/A	2033	1211	61.49	77.03	81.76	82.43 (7)
RKB	Human Annotated	893	0	3244	1865	66.89	**84.46**	89.86	90.54
Easy Inst.	ESX	2469	1576	1026	570	47.30	57.78	61.49 (3)	-
Easy Inst.	ELX	3522	2629	565	303	32.43	39.86	41.22	-
Hard Inst.	HSX	1101	208	**3712**	**2279**	70.95	**84.46**	**91.22**	**91.89 (6)**
Hard Inst.	HLX	1228	335	3437	2107	**72.30**	**84.46**	88.51	89.96 (5)
Results from ablation of the keypoint rectifier during evaluation									
Hard Inst.	HSX	1101	208	3270	2024	69.59	83.11	90.54	91.22 (5)
Hard Inst.	HLX	1228	335	2827	1734	66.89	82.43	86.49	87.16 (5)

## Data Availability

Dataset available on request from the author, Amy R. Reibman.

## Appendix A. Details of Model Training

We trained the keypoint-RCNN model with the R101-FPN backbone using the Detectron2 framework on Nvidia A6000 GPUs (NVidia, Santa Clara, CA, USA) with a batch size of 6. All default models in half-day experiments are trained for 30,000 iterations. They are then evaluated on the test set videos to produce easy and hard instances. The easy and hard instances are then collected and used to form the expanded datasets. The training of the models on these expanded datasets starts again from ImageNet pretrained weights and goes on for 30,000 more iterations. In exactly the same way, for whole-day experiments, all the models are trained for 50,000 iterations. The models of the EI2023 baseline were also trained for 50,000 iterations in [1]. We chose to train with images at their original resolution of 1920 × 1080 px instead of scaling them down to preserve instance mask and keypoint annotation quality.

## Appendix B. Detailed Results of Half-Day Experiments

In this section, we present the results of half-day experiments in Table A1. The values in this table are the values used in the plots in Figure 10 and Figure 11.

**Table A1:** Results for half-day experiments. The results in this table use the derived evaluation metrics. The best model is the one with the highest number of correct localizations, identifications, and video-level top-K accuracy for the highest possible K value. The highest value in each evaluation column is highlighted in bold.

	Training Dataset Information Day1-SetA			Evaluation Information					
				Fresh Day (Total)			Unseen Cows (Average)		
Experiment Info	Dataset Name	#Training Images	#Easy/Hard Inst. Used	#Correct Localizations	#Correct Identifications	Top-Max Accuracy (%)	#Correct Localizations	#Correct Identifications	Top-Max Accuracy (%)
RKB	$D^{\prime }$	76	0	639	386	49.32	322	198	49.33
Easy Inst.	$ESX^{\prime }$	112	36	767	515	58.11	407	274	60.00
Easy Inst.	$ELX^{\prime }$	155	79	736	491	53.38	438	312	62.00
Hard Inst.	$HSX^{\prime }$	143	67	943	618	59.46	495	335	64.67
Hard Inst.	$HLX^{\prime }$	196	120	**1301**	**795**	**70.27**	**738**	**464**	**72.00**
